# Using the Practical, Robust Implementation and Sustainability Model to inform meaningful partner engagement and the selection of implementation strategies to increase colorectal cancer screening in federally qualified health centers

**DOI:** 10.3389/fpubh.2025.1617770

**Published:** 2025-10-16

**Authors:** Jesse Nodora, Samir Gupta, Samantha Hurst, Aimee S. James, Borsika A. Rabin

**Affiliations:** ^1^Department of Radiation Medicine and Applied Sciences, Community Outreach and Engagement, UC San Diego Moores Cancer Center, University of California, San Diego, San Diego, CA, United States; ^2^Division of Gastroenterology, Department of Internal Medicine, Moores Cancer Center, University of California, San Diego, San Diego, CA, United States; ^3^Herbert Wertheim School of Public Health and Human Longevity Science, University of California, San Diego, San Diego, CA, United States; ^4^Division of Public Health Sciences, Washington University in St. Louis, St. Louis, MO, United States; ^5^Herbert Wertheim School of Public Health and Human Longevity Science, UC San Diego Altman Clinical and Translational Research Institute, Dissemination and Implementation Science Center, University of California, San Diego, San Diego, CA, United States

**Keywords:** pragmatic robust implementation and sustainability model, RE-AIM, colorectal cancer screening, underserved communities, implementation strategies

## Abstract

Engaging community partners in research meaningfully can guide effective implementation efforts. This approach is particularly crucial when we work with complex, multilevel programs in low-resource settings that serve diverse populations. The application of dissemination and implementation science theories, models, and frameworks to facilitate the iterative, multilevel engagement of partners in selecting and optimizing implementation strategies is not commonly described in the literature. In collaboration with three federally qualified health centers in San Diego County, we utilized the Practical, Robust Implementation and Sustainability Model (PRISM), which is a contextually expanded version of the widely used Reach, Effectiveness, Adoption, Implementation, and Maintenance (RE-AIM) framework, to guide partner-engaged data collection on processes, resources, facilitators, and barriers for colorectal cancer (CRC) screening. We gathered implementation-relevant information from each FQHC, including partner introductory meetings, an Agile Science workshop, secondary data collection, surveys, and in-depth interviews. Insights from the PRISM domains led to the development of process maps that guided the selection of implementation strategies to support the use of evidence-based interventions for CRC screening.

## Background

Improvement of colorectal cancer (CRC) screening uptake, particularly in low-resource settings, is necessary for reducing the incidence of advanced CRC among traditionally-underserved populations. The success of sustainable evidence-based interventions (EBI) for CRC screening in these settings varies despite the availability of implementation strategies and practice-oriented resources. At the patient level, these interventions include reminders, small media, and one-on-one education. For providers these include assessments and feedback on their CRC screening performance and the use of reminder and recall systems (e.g., tablet info linked to patient medical record). At the clinic-level, EBIs focus on the reduction of structural barriers such as expanded clinic hours and ease of access to tests and results (1). In federally qualified health centers (FQHCs), CRC EBIs are often partially or intermittently implemented (2, 3), and sustainability of these programs would improve CRC screening rates.

Several barriers to implementing such programs at FQHCs have been identified and include patient knowledge of and access to CRC screening, staff time and resources for efficiently identifying patients due for screening, and the degree to which EBIs align with institutional culture and the target population (4). In addition, factors such as number of clinic personnel and accuracy of electronic medical records have also been shown to impact screening (5), as well as the presence of a wide variety of healthcare priorities, unavailability of federal funding for educating patients, and a dearth of funding for follow-up care for patients who have a positive screen (6). Consequently, FQHCs have notably lower CRC screening rates (41%) compared to the American Cancer Society National Colorectal Cancer Roundtable screening target of 80% (7). Rigorous approaches to improve these rates include the application of dissemination and implementation (D&I) scientific methods (8, 9), which emphasize the importance of understanding unique contextual factors that influence effective and sustainable implementation of EBIs in practice settings (10). Further, evidence from prior studies demonstrates that collecting data using meaningful partner engagement, guided by D&I theories, models, and frameworks (TMFs), may improve the use of EBIs and allow for more effective selection of implementation strategies in FQHC settings (2, 11).

The Practical, Robust Implementation and Sustainability Model (PRISM) (12–15) aims to identify EBI contextual factors and related implementation strategy selection and application. PRISM expands the updated (Glasgow, Harden et al.). Reach, Effectiveness, Adoption, Implementation, Maintenance (RE-AIM) framework with a multilevel contextual understanding, including considerations for the intervention, recipients, implementation and sustainability infrastructure, and external environment from the organizational and patient/community member perspectives. PRISM is well suited to guide multilevel data collection from multilevel partners as well as contextual data underpinning organizational capacities for EBI and implementation strategy selection in FQHC settings. Several previous studies used PRISM and partner engagement approaches to assess diverse partner perspectives and feedback to inform EBI implementation (13, 15–19). Most notably, Fort discussed how to use PRISM with an equity lens and Perez Jolles provided a clear application of the use of PRISM for co-creation with partners (14). Additional work on how to use RE-AIM and PRISM to improve the alignment of the intervention and context has been published by Glasgow et al. (20–22) and Trinkley et al. (23).

This manuscript presents our data collection process for identifying implementation strategies at FQHCs, which was guided by PRISM. We provide results on the external and internal contextual factors that may influence the selection and implementation of EBIs in an FQHC clinical setting. We also present a visual representation of a process map of the CRC screening process at one participating FQHC which was generated from this work.

## Study design and methods

### Project FACtS

Project FACtS (FQHCs Assessing Colorectal cancer Screening) is an American Cancer Society funded D&I study (Nodora J, PI) that builds on an established and integrated clinical-academic partnership with three San Diego FQHCs serving predominantly low income, publicly and/or non-insured Hispanic/Latino patients. The overall purpose of the FACtS study is to use quantitative and qualitative data from diverse partners to develop process maps that inform the selection and adaptation of EBIs and implementation strategies using the United States Community Preventive Services Task force (CPSTF) interventions (1) and the National Colorectal Cancer Roundtable (NCCRT) “Steps Manual” (24) strategies for CRC screening. Based on their current prevention practices and needs, participating FQHCs will implement CPSTF EBIs (e.g., reducing structural barriers at the clinic level, provider assessment and feedback, and patient reminders and one–one education) and relevant NCCRT implementation strategies (i.e., make a plan, assemble a team, screen patients, coordinate care) to increase their CRC screening.

Project FACtS applies PRISM (multilevel PRISM context domains and RE-AIM outcomes) to assess the relevant internal and external environments and their fit with the characteristics of EBIs for CRC screening. Internal environmental factors included consideration of service recipients and implementation and sustainability infrastructure at each FQHC, while external variables may include available community resources and policy impacts.

**Figure 1:**
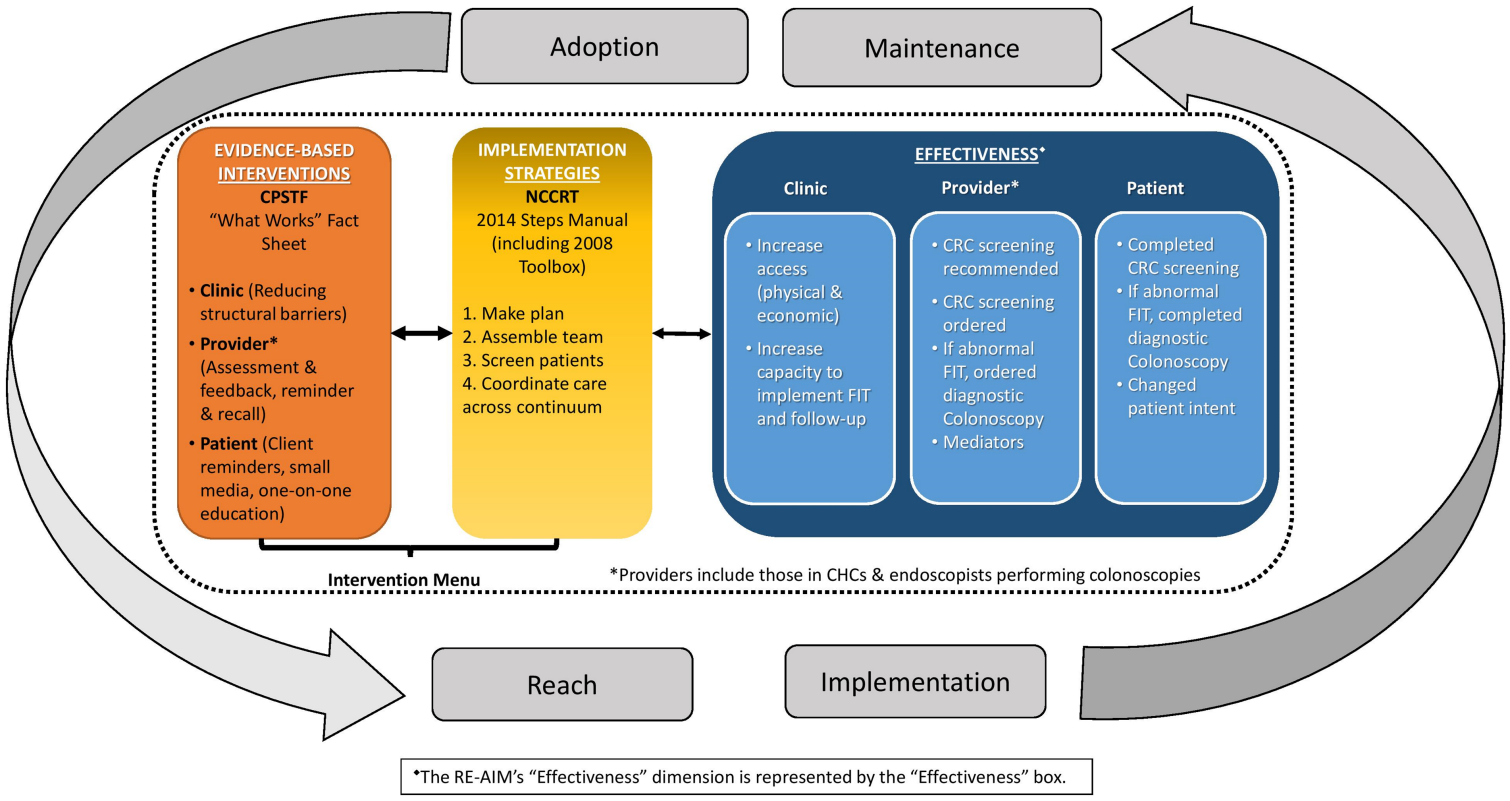
Conceptual model for increasing colorectal cancer (CRC) screening among populations in community health center (CHC) settings.

Figure 1 presents the Project FACtS conceptual model. The four model elements are: (1) CPSTF EBIs; (2) NCCRT implementation strategies; (3) multi-level outcomes (i.e., process and effectiveness measured at clinic, provider, and patient level); and (4) RE-AIM dimensions (i.e., reach, adoption, implementation, and maintenance). The first two elements include CRC screening EBIs and implementation strategies available to FQHCs for future intervention. The bidirectional arrows indicate a relationship among interventions, strategies, and outcomes. As EBIs and implementation strategies are implemented and their outcomes assessed, relevant findings may influence and modify the model. Finally, the bidirectional arrows from the RE-AIM dimensions show how these may influence the selection of EBIs, implementation strategies, and the outcomes (effectiveness).

### Community partners

Partner engagement with our three Project FACtS FQHC partners began 3 to 4 years before the work described herein commenced. Through various interactions, including networking meetings, pilot data collection, and dissemination and dialogue of pilot fundings we applied the Principles of Community Engagement (U. S. Department of Health and Human Services 2011), specifically: becoming knowledgeable about each FQHC, establishing relationships and trust, exploring how community-academic partnerships can improve health and build mutual capacity, respecting FQHC self-determination and diversity, and working toward a long-term commitment.

The community-academic partnership in Project FACtS includes sustained interaction among clinic-based coordinators, senior quality improvement leads, the FQHC executive teams, and our University of California San Diego research team. Our community partners are established FQHCs that provide care to medically underserved, racial/ethnic minorities, and low-income populations. Our partner sites’ 2021 CRC screening rates were 37, 51, and 66%, with varying levels of CRC-specific grant funding, outreach, and programming to increase screening (25). Project FACtS allocated resources for each of the three FQHCs to hire a clinic-based study coordinator to manage project tasks. This position was specifically created to reduce clinic staff burden during the study period. The three clinic-based coordinators work with an academic-based study coordinator who serves as a liaison between the study’s FQHC partners and academic team. The protocol (#180364X) for this study was approved by the University of California, San Diego Institutional Review Board.

### Data collection

We used multiple consecutive and iterative data collection approaches guided by PRISM to gather information about implementation-relevant information from each FQHC, including partner introductory meetings, an Agile Science workshop, secondary data collection, surveys, and in-depth interviews (Table 1). Each data collection approach was informed by the paradigm of meaningful partner engagement and increased in specificity. As such, data collection was iterative, and findings from each previous approach informed each subsequent approach. The groundwork laid by findings from meetings, the Agile Science workshop, and secondary data collection reduced participant burden by shortening later surveys and in-depth interviews, and produced rich, largely partner-driven results. Because this manuscript focuses on the use of PRISM to guide meaningful partner engagement for the selection of implementation strategies, detailed methods and study results, particularly for the surveys and in-depth interviews, are beyond the focus of this manuscript. These will be presented in future manuscripts.

**Table 1:** Partner engagement and data collection approaches.

Data collection method	Partner participants	Objectives
Introductory meetings	Clinic coordinators, lead quality improvement specialist, CEO, CMO, physician champions, lab personnel and data analysts, and academic team	Develop priorities; elicit feedback on study design and data collection; set guiding principles; decide on outcome measures
Agile science workshop	Agile Science team, Site-based coordinators, Quality improvement specialists, and academic team	Inform data collection processes; discuss feasible site-specific strategies to improve CRC screening; conduct preliminary mapping of CRC screening at each FQHC
Coordinator secondary data requests	Site-based Coordinators	Collect data for internal clinic variables such as clinical characteristics and screening rates; and for external influences, data such as CRC-specific policies in health plans, FQHC accrediting organizations; FQHC funders, and CRC-linked organizations
Online surveys	CEO, medical director, primary care providers, clinic managers, referral specialists, quality improvement specialists, and gastroenterologists	Collect data regarding patient-level barriers for CRC screening, the referral process for diagnostic colonoscopy following abnormal FITs, and existing referral GI and FQHC relationships
In-depth interviews	CEO, medical director, clinic manager, lab manager, and quality improvement specialist	Gather detailed information that emerged as critical from the previous data collection methods, particularly the surveys

#### Introductory meetings

The study team held introductory in-person meetings with each FQHC Project FACtS team. These meetings lasted between 60 and 90 min and took place at each FQHC. Participants included a diverse array of partners such as the Chief Executive Officer, Chief Medical Officer, head of Quality Improvement, clinic coordinator, physician champions, and other clinical and quality service staff (e.g., lab personnel and data analysts). The primary goal of these meetings was to establish a collaborative working relationship between the research team and health center staff. Specific objectives included developing a shared understanding of priorities and ongoing activities undertaken by the FQHC for CRC screening, as well as gathering feedback on the proposed study design, data collection methods, and measures. Additionally, we discussed the main components for the guiding principles of our study partnership. Notes from various research team participants and follow-up team meetings were compiled to inform the design, data collection, and decisions regarding outcome measures for subsequent phases.

#### Agile science workshop

**Figure 2:**
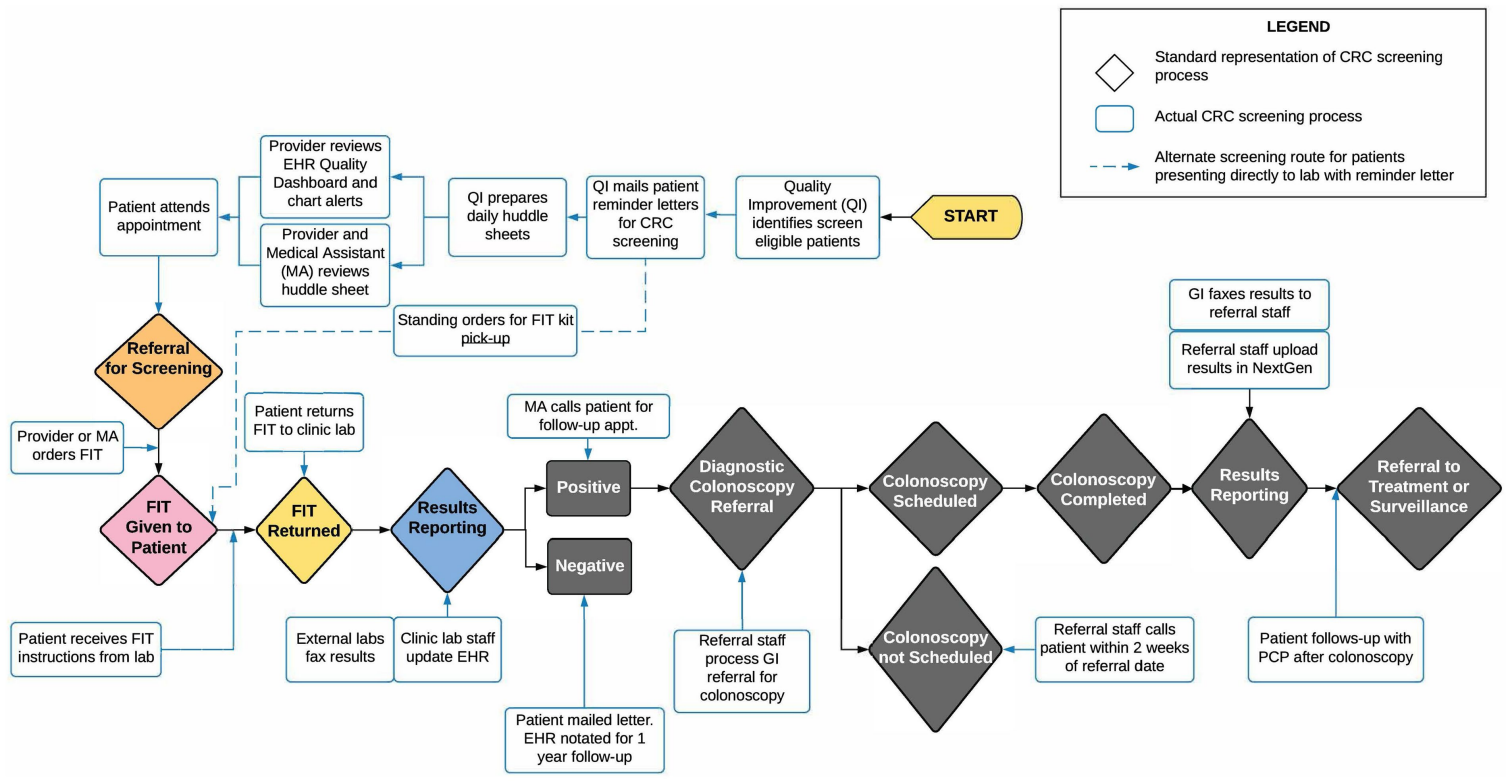
Sample process map for CRC screening by FOBT/FIT at one FQHC.

Using an Agile Science http://www.agilescience.org/ approach (26), preparatory meetings and a half-day workshop were held with FQHC partners. Agile Science is an innovative methodology that was designed to engage in meaningful partnerships across diverse partners with the goal of rigorously, yet efficiently, testing assumptions about how a process unfolds to achieve any particular outcome, such as how CRC screening practices are implemented with the ultimate goal of improving screening rates at FQHCs. Core to the Agile Science methodology is the recognition that people are different, context matters, and both often change. This multi-disciplinary approach was applied to inform data collection processes, discuss feasible strategies to improve CRC screening, and to conduct preliminary mapping of the CRC screening process at the participating FQHCs and their clinic sites. This approach contributed to active partner engagement and the early development of CRC screening process maps (See Figure 2).

The Agile Science process employed six steps—align, explore, create pre-workshop, create workshop, reflect, and post-workshop evaluation—over a seven-month period. The initial steps (align and explore) defined workshop goals and aligned relevant study outcomes with Agile Science concepts. Following that, the Agile Science team (external consultants to the research team) conducted six 20–30 min pre-workshop telephone interviews with seven FQHC staff to inform the final design of the 4-h workshop. The workshop was attended by six staff from the three FQHCs, the Agile Science team, and our research team. Activities built upon the pre-workshop interviews and involved initial mapping of CRC screening processes and preliminary identification of intervention points at each FQHC. Results were documented as notes and draft process maps. An evaluation survey administered to participants afterward assessed workshop usefulness and satisfaction, including feedback on areas of the CRC screening process that our partners expressed interest in further exploring.

#### Secondary data

Secondary data used for this work included internal clinic data such as clinic characteristics and CRC screening rates and processes, as well as external CRC-relevant influences arising from outside the FQHC system. We consider this “secondary data” because we asked one person at each FQHC (our clinic-based coordinator) to respond using existing information from prior assessment or evaluation reports. For internal data, clinic characteristics and screening rates were extracted from 2018 Uniform Data Systems (UDS) collected by the federal Health Resources and Services Administration (HRSA), an agency of the U. S. Department of Health and Human Services which funds FQHCs (25). Contextual clinic data for CRC screening processes were gathered using a “Secondary Data Request Form.” This form consisted of 29 questions developed by the research team, with input from FQHC partners, and was organized by PRISM contextual domains to collect preliminary information on the factors influencing CRC screening. Examples of the questions included: Using a list of CPSTF EBIs, “…which approach would work best for patients and providers at (your FQHC) for colorectal cancer (CRC) screening?” and “Describe (your) organizational structure for CRC screening.” The data request form was completed by each clinic-based coordinator for their respective FQHC and served as the baseline for documenting CRC screening processes for each FQHC system.

For external influences, data were collected on CRC-specific policies in health plans for both government (Medicaid and Medicare) and private (e.g., Molina Healthcare) carriers; CRC-specific information from FQHC accrediting organizations (National Committee for Quality Assurance-NCQA); CRC-tracking and quality requirements from FQHC funders (Health Resources and Services Administration-HRSA); and CRC-linked organizations (American Cancer Society-ACS, California CRC Coalition-C4). These data were collected from websites and existing reports.

#### Surveys

Surveys were conducted online using Qualtrics software (Qualtrics, Provo, UT) for FQHC leadership and clinical staff. The development of the survey was informed by the various PRISM domains and refined through partner input and secondary data, aiming to gather essential information on patient-level barriers to CRC screening, the referral process for diagnostic colonoscopy following abnormal FITs, and existing referral relationships between GI specialists and FQHCs. Partners involved in the survey development and feedback on the initial items included at least five key personnel from each FQHC. These participants included the executive director, medical director, quality improvement specialists, clinic managers, referral specialists, primary care providers, and gastroenterologists to whom the three FQHCs refer patients. The coordinators at the clinics invited their respective staff to participate in the survey and oversaw survey reminders and follow-ups.

#### In-depth interviews

The introductory meetings, Agile Science workshop, and surveys informed the development of in-depth interview guides, which were developed around the PRISM domains and refined using previously-collected data. The interviews were designed to gather detailed information that emerged as critical from the previous data collection methods, particularly the surveys. Like the surveys, interviews were conducted with at least five key clinic personnel including the executive director, medical director, quality improvement specialists, clinic manager(s), and lab manager(s). Interviews were conducted at all participating FQHCs for the listed key personnel, 5 per site (15 total). Interview guides were tailored to each participant based on their primary role. All interviews were consented, audio-recorded, transcribed, and were completed in less than an hour. The clinic and academic-based coordinators scheduled the interviews and provided logistical support during the interview phase. All interviews were conducted by trained interviewers.

### Analysis and results synthesis (for process map)

Notes from the introductory meetings were compiled and organized by PRISM domains and focus areas including design, methods, and measures. Guiding principles were drafted and further refined after iterative interaction with each FQHC. Data from the Agile Science workshop were summarized as action items and draft process maps were recorded and used to provide initial drafts of an FQHC-specific process map. Secondary data were reviewed, qualitatively summarized, and incorporated into subsequent data collection. Survey data were extracted from Qualtrics and analyzed using descriptive statistics. The research team conducted the first iteration of data analysis and a summary report was prepared for each FQHC describing the CRC screening process and influential contextual factors. Partner meetings were held to review the data reports for accuracy and completeness.

### Process map development

Information from multiple waves of data collection was used to inform the development of CRC screening process maps, developed with Lucid Chart Software, using methodology adapted from James et al. (Manuscript in preparation). These maps were developed as a tool to visualize the CRC screening process for each FQHC and facilitate the identification of CRC screening intervention points. During process mapping, the study team met frequently with FQHC partners to discuss accuracy and elicit feedback, which was then incorporated until the maps were deemed to be complete.

## Findings

Table 2 presents the findings for preliminary data collection organized by PRISM internal and external domains. For partner engagement/PRISM External Context, the introductory team meetings and Agile Science workshop provided useful insights on external influences on CRC screening, including relevance of health plan coverage and importance of both funding (from health plans and HRSA) and accrediting (e.g., NCQA) organizations on the collection of CRC screening rates guided by the Patient-Centered Medical Home and the Safety Net Medical Home Initiative (27). For Internal Context, preliminary findings from our introductory full team meetings revealed that our engagement approach was feasible and well-received by all participants. Partner engagement was excellent with a range of 4–13 participants from each FQHC attending introductory team meetings. Insights from interviews with community health centers partners included that there was “v*alue in getting to a point where we can translate assumptions as articulated through causal diagrams (the influence maps) into measures of success* (i.e.*, operationalizing proximal outcomes*)*,”* and to be mindful of terminology used throughout the project, as Agile Science approaches and the D&I project employed different terms; as such, participants found it challenging to “translate” the wording from one methodology to the other. Respondents highlighted that it was important for the success of the process to have included the involvement of community partners from the start of the project. They recommended that the research team should consider, “*being very thoughtful about what works for each clinic and tailoring to those needs and ensuring the center can maintain practices on their own*.”

**Table 2:** Findings by various data collection approaches organized by PRISM domains.

Data collection approach	PRISM domains	Key findings
Partner engagement approach		
Introductory meetings	External context External environment	*Health Plans*: Medicare and Medicaid (Medi-Cal) CRC screening coverage. Some health plans offer incentives to FQHCs when screening metrics are met.
	Internal context Multi-level organizational & patient characteristics Multi-level organizational perspectives Implementation & sustainability infrastructure	*Meeting attendance*: Meetings were all well attended at all 3 participating FQHCs. *Organizational staff structure*: Participating FQHC staff include the executive team (CEO and CMO), Quality Improvement team (director, specialists, and analysts), provider champion, and CHC-study coordinator. *Values and priorities*: FQHCs committed to improving cancer screenings and EHR systems. *Current CRC screening challenges*: EHR capacity to provider EHR reminders, team feedback on returned FIT kits.
Agile Science workshop	Internal context Multi-level organizational & patient characteristics Multi-level organizational perspectives Implementation & sustainability infrastructure	*Satisfaction*: Partners were satisfied with the workshop experience, with a mean reported score of 4.33 (based on 5-point Likert scale). Participants affirmed value in having time to discuss interventions in small groups with a mix of staff across all 3 FQHCs. *Acceptability*: The workshop activated an inclination to cross-collaborate with other local FQHCs to brainstorm CRC screening interventions. *Usability*: The draft process maps produced in the workshop set the tone for developing formal CRC screening process maps at each FQHC. All participants wanted to explore potential points for “interventions” based on identified weak and strong points along the screening process (i.e., via process maps).
Data collection approach		
Secondary data*	External context External environment	*Reporting*: FQHCs report CRC screening rates to NCQA (for PCMH and HEDIS), HRSA. One FQHC also reports to CDPH. *External Labs*: FQHCs partner with external labs to process FIT kits. This approach works well and FQHCs did not report any significant issues. *Grants & Partnerships*: FQHCs receive, or have received, external grant funding from C4 to increase CRC screening. FQHCs also collaborate with other research institutions and local organizations. *Health Plans*: FQHCs accept majority of plans. One FQHC experiences variation in coverage for FIT test brands depending on the health plan and affiliated lab. For the same FQHC, pre-authorizations for colonoscopy may be a clinical and patient barrier.
	Internal context Multi-level organizational & patient characteristics Multi-level organizational perspectives Implementation & sustainability infrastructure	*Patient demographics*: FQHCs primarily provide care for racial/ethnic minorities, uninsured or underinsured, and low-income populations. *Clinic demographics*: FQHCs have 4 to 8 clinic sites in San Diego County. *Organizational structure for CRC*: The Quality Improvement Dept. consists of QI Specialists, data analysts and manages reports and improvement efforts to increase screening. The clinic site leadership (i.e., clinical team leader and site manager) and the providers and care team are also involved in the CRC screening process. FQHCs also have HEDIS teams that conduct patient outreach. *CRC screening approaches currently in place*: FQHCs are currently implementing a combination of: Patient reminders, small media, one-on-one education, reducing structural barriers, provider assessment & feedback, provider reminders & recall, *CRC screening challenges*: For one FQHC, challenges exist in EHR capacity for managing GI referrals, lack of workflow for uninsured patients, documenting screened patients to determine baseline screening rates. For the majority of FQHC’s, challenges exist for provider assessment and feedback. *EHR capacity*: FQHCs use NextGen or eClinicalWorks systems for medical records. Existing capacity to identify age-eligible unscreened patients and patients with abnormal FIT with completed colonoscopy. Existing capacity to run CRC screening reports using EHR system and/or combined use of i2i Tracks or other customized reporting software (Bridge IT, SQL/C# codes)
Surveys	External context External environment	*GI Referral Relationship*: Referral agreements not in place between GIs and FQHCs. Patients are referred to GIs based on distance and accepted health plans. *GI Referral Challenges*: Existing challenges in the delivery of colonoscopy reports from GI to FQHC, and from FQHC’s referrals dept. to provider. Scheduling challenges due to limited appt. Availability for colonoscopy.
	Internal context Multi-level organizational & patient characteristics Multi-level organizational perspectives Implementation & sustainability infrastructure	*GI Referral Process (internally):* Patients with an abnormal FIT are referred to a GI specialist for colonoscopy. The FQHC referrals dept. processes the referral request and follows-up with patients to schedule colonoscopy. Patient follows-up with PCP post-colonoscopy. (See Figure 2). *Patient CRC screening barriers*: Long wait times of 2 to 3 months, lack of insurance, colonoscopy prep education, difficulty reaching patient, language barriers, transportation, and lost wages
In-depth interviews	External context External environment	*Expected Learnings*: External support (i.e., reporting and accrediting agencies) for CRC screening approaches currently in place.
	Internal context Multi-level organizational & patient characteristics Multi-level organizational perspectives Implementation & sustainability infrastructure	*Expected Learnings:* Decision-making, implementation process, evaluation and reporting, and organizational support (i.e., leadership, providers, and staff) for CRC screening approaches currently in place.

*Assessed from the Uniform Data System (UDS), a reporting system within the Health Resources Services Administration (HRSA), and CHC secondary data requests.

NCQA, National Committee for Quality Assurance; PCMH, Patient Centered Medical Home; HEDIS, Healthcare Effectiveness Data and Information Set; HRSA, Health Resources Services Administration; C4, The California Colorectal Cancer Coalition; CEO, Chief Executive Officer; CMO, Chief Medical Officer; GI, Gastroenterologist; EHR, Electronic Health Record; CDPH, California Dept of Public Health.

The results of the Agile Science post-workshop evaluation, which were most relevant to the PRISM Internal Context, showed that participants affirmed value in discussions grounded in prior knowledge, having time to discuss interventions, using small group breakouts, and getting everyone together in one room. Participants indicated that they would prefer more guidance for the various exercises as well as concrete next steps. If more time were available during the workshop, all participants wanted to explore potential points for “intervention” based on identified weaknesses and strengths along the screening process. Specific responses included: “*being able to identify a process for improvement that could increase compliance rates*”; “*maybe uncover a different approach, when a “bad node” is blocking the path*”; “*determine impact we could have in those areas and best practices*”; and “*change/adjust what we initially planned - take concrete steps to adjust to what we heard during the workshop*.”

Key findings by PRISM domains for our coordinator secondary data requests, online surveys, and in-depth interviews are also presented in Table 2. The secondary data requests yielded key external context information, such as partnerships with laboratories for processing FIT tests, how FQHCs have been funded to increase CRC screening, and which health plans are accepted. For internal context, we gathered data for the demographics of the population served by each FQHC, the number and location of clinic sites, and the organizational structure for CRC screening processes.

Administration of online surveys provided additional information regarding external context factors, including how agreements for referrals to gastroenterologists after screening are executed and how data from these procedures are communicated back to the clinics. The surveys also identified barriers, such as limited appointment availability for colonoscopies. Internal context included extensive data about clinic follow-up after a positive screening test and further details on screening barriers, such as wait times for colonoscopies. Finally, in-depth interviews revealed critical insights about the level of external support for existing CRC screening processes, as well as clarifying steps within the clinics related to CRC screening, including implementation procedures and internal organizational support.

### Findings for process mapping

Using the evaluation results from the Agile Science workshop, secondary data results, and provider surveys, we constructed preliminary process maps. Figure 2 displays an example of one such map; creating tailored maps for each FQHC uncovered variation in the CRC screening processes among different FQHC systems. The visual representation of the CRC screening process in the process map enabled participating FQHCs to pinpoint specific intervention points within their distinct CRC screening process. In preparation for intervention testing, each map can aid in selecting implementation strategies and suitable EBIs.

## Implications for D&I research

The purpose of this work was to employ PRISM to guide a meaningful partner engagement approach to develop FQHC process maps with the overall goal to facilitate EBI selection, adaptation, and implementation for CRC screening in individual FQHC settings. The use of PRISM in this process was both relevant and practical, providing a potential application for future D&I studies in low-resource primary care settings. The work described in this manuscript helped shape the role of context as it is defined by the PRISM three domains: *characteristics of the intervention and recipient* as perceived by diverse partners; *Implementation and sustainability infrastructure*; and *External environment* (21). We identified external and internal contextual factors that may influence implementation of EBIs in an FQHC clinical setting. Through tailored process maps, we provided visual representations of the CRC screening process for each participating FQHC. These maps will ultimately be used to guide the FQHC’s selection of implementation strategies and EBIs that will be most effective and efficient within each FQHC.

McCreight et al. studied the impact of using PRISM to identify barriers and facilitators to implementation of interventions. They found it to be a useful framework for understanding context around content areas and along the various steps in program implementation (17). The authors concluded that it is critical to continue assessing PRISM’s ability to identify and guide the measurement of the contextual factors that emerge as most important for successful implementation, and for which outcomes they pertain. The work herein adds to the body of literature demonstrating the successful use of an iterative process built on PRISM to identify such factors and tailor them to individual FQHCs.

In preparation for a randomized trial, PRISM provided a useful organizing framework to gather information on existing CRC screening processes, capturing robust data that indicated where improvements could be made, and identification of which EBIs may create these improvements. We used PRISM to develop measurement instruments, including meeting agendas, secondary data collection, surveys, and interview guides; to cross-validate instruments such as the interview guide and survey questions, and in data analysis and organization of findings. Specifically, for instrument development, we aligned questions with each PRISM domain to ensure we captured all critical context areas. Partner input and information from prior rounds of data collection helped us refine the initial questions and focus them on emerging domains that seemed most relevant in a given context. A major benefit of the use of an overarching model was that it allowed our team to link the various data collection waves through constant validation of the PRISM domains. Use of PRISM also allowed for cross-FQHC comparisons in processes and to identify key similarities and differences between these sites. These comparisons can inform the tailoring and refinement of the implementation strategies used in this study. Finally, PRISM was a natural organizer of our data analysis efforts and allowed us to display our findings efficiently while still maintaining fidelity to our theoretical model.

The strengths of this work include the rigorous, thorough, multi-step process for identification of individual CRC screening program implementation needs for each FQHC. Limitations included that the study was limited to three FQHCs in the San Diego area, which may restrict the application to diverse FQHCs. While PRISM was found to be useful and feasible across the early stages of this project, we also acknowledge several opportunities for improvement.

Operationalization of implementation science TMFs is most effective when team members have expertise in their specific use. Additionally, while TMFs can be meaningful for a research team, they are sometimes less meaningful to practitioners such as clinical and community partners. It is important to avoid jargon and gather information about each domain in the TMF in a way that is easily understood and meaningful for the various partners. A recent resource, the iPRISM web tool^1^, was developed based on PRISM and provides real-time definitions and examples to create meaning for each PRISM domain while guiding users through the assessment process. Using multiple, increasingly specific, methods can lead to added burden for partners who are invited to on-going and/or iterative data collection efforts. Finding the “sweet spot” for data collection is critical where the burden is not overwhelming, and partners appreciate the value of data collection efforts and are not burdened by the time and effort. In our study, partners expressed a sense of co-ownership over the project which increased their interest and willingness to contribute. Co-creation has been increasingly described as a desirable approach in informing intervention and implementation strategy development (28).

Use of TMFs has a long history in intervention science and health behavior change, and is becoming routine practice in implementation science. In this study, we were able to demonstrate how to operationalize one model (PRISM) in a longitudinal, iterative manner and benefit from the shared constructs across timepoints, data collection and analytic approaches, and locations. Future studies should consider implementation science TMFs, applying these throughout the study’s lifetime to inform research questions, design, data collection and analysis, and the interpretation, synthesis, and dissemination of findings. Furthermore, use of multiple strategies to gather information from multiple partners to increase the alignment of interventions and implementation strategies with the local context can be a beneficial approach to prepare for larger experimental implementation studies. In summary, the present work demonstrated the usefulness of a thorough, rigorous PRISM-guided process for the identification of factors impacting the implementation of CRC screening programs within individual FQHCs. This research resulted in the creation of process maps as guides for each FQHC to use for future implementation of CRC screening interventions, adding to the literature on the effectiveness of PRISM for these tasks. Future work should assess the impact of these tailored interventions on program sustainability and success rates as measured by increased CRC screening.

## Data Availability

The datasets presented in this article are not readily available because qualitative and secondary data are not available to public.

## Footnotes

^1^ https://prismtool.org/
